# Omega-3 fatty acids correlate with gut microbiome diversity and production of N-carbamylglutamate in middle aged and elderly women

**DOI:** 10.1038/s41598-017-10382-2

**Published:** 2017-09-11

**Authors:** Cristina Menni, Jonas Zierer, Tess Pallister, Matthew A. Jackson, Tao Long, Robert P. Mohney, Claire J. Steves, Tim D. Spector, Ana M. Valdes

**Affiliations:** 1 Department of Twin Research, King’s College London, London, UK; 2 Institute of Bioinformatics and Systems Biology, Helmholtz Zentrum München, Neuherberg, Germany; 3 Sanford Burnham Prebys, La Jolla, USA; 4 Metabolon Inc., Research Triangle Park, NC 27709 USA; 5 School of Medicine, Nottingham City Hospital, Hucknall Road, Nottingham, UK; 6 NIHR Nottingham Biomedical Research Centre, Nottingham, UK

## Abstract

Omega-3 fatty acids may influence human physiological parameters in part by affecting the gut microbiome. The aim of this study was to investigate the links between omega-3 fatty acids, gut microbiome diversity and composition and faecal metabolomic profiles in middle aged and elderly women. We analysed data from 876 twins with 16S microbiome data and DHA, total omega-3, and other circulating fatty acids. Estimated food intake of omega-3 fatty acids were obtained from food frequency questionnaires. Both total omega-3and DHA serum levels were significantly correlated with microbiome alpha diversity (Shannon index) after adjusting for confounders (DHA Beta(SE) = 0.13(0.04), P = 0.0006 total omega-3: 0.13(0.04), P = 0.001). These associations remained significant after adjusting for dietary fibre intake. We found even stronger associations between DHA and 38 operational taxonomic units (OTUs), the strongest ones being with OTUs from the *Lachnospiraceae* family (Beta(SE) = 0.13(0.03), P = 8 × 10^−7^). Some of the associations with gut bacterial OTUs appear to be mediated by the abundance of the faecal metabolite N-carbamylglutamate. Our data indicate a link between omega-3 circulating levels/intake and microbiome composition independent of dietary fibre intake, particularly with bacteria of the *Lachnospiraceae* family. These data suggest the potential use of omega-3 supplementation to improve the microbiome composition.

## Introduction

There is evidence indicating that dietary supplementation with omega-3 polyunsaturated fatty acids (PUFA) may improves some health parameters in humans^1^. Docosahexaenoic acid (DHA) is an omega-3 fatty acid that is a main structural component of the human brain, cerebral cortex, skin, sperm, testicles and retina^2^. Higher circulating levels of DHA are associated with lower risk of future cardiovascular events in three prospective population based cohorts^3^. The other main omega-3 fatty acid is eicosapentaenoic acid or EPA, and omega-3 levels in humans are estimated by the sum of EPA + DHA with docosapentaenoic acid (DPA) being present at much lower concentrations^4^. Positive effects on health from omega-3 fatty acids have been observed for insulin resistance, adult-onset diabetes mellitus^5–7^, hypertension^8, 9^ arthritis^10, 11^, atherosclerosis^12, 13^, depression^14, 15^, thrombosis^16^, some cancers^17^ and cognitive decline^18, 19^.

These fatty acids can only be synthesized in mammals from the dietary precursor and essential fatty acid, α-linolenic acid^1^. However, the synthesis pathway requires a number of elongation and desaturation steps, making direct uptake from the diet a more effective route of assimilation. EPA and DHA in the human diet are derived primarily from marine algae (higher plants lack the enzymes for the biosynthesis of these lipids), which is concentrated in the flesh of marine fish where bioavailability is dramatically increased^20^. Some of the mechanisms whereby omega-3 fatty acids operate are linked directly to their anti-inflammatory actions since both EPA and DHA decrease synthesis of the pro-inflammatory prostaglandin E2^21^. EPA and DHA are also precursors of the E-resolvins and D-resolvins that suppress inflammatory cytokine production and act to resolve inflammation^22^.

There is some evidence from case reports and from animal studies suggesting that the effect of omega-3 on the gut microbiota may also play an important role in the effects of omega-3 polyunsaturated acids on clinical parameters^23–25^. The relationship between the gut microbiota and its host plays a key role in immune system maturation, food digestion, drug metabolism, detoxification, vitamin production, and prevention of pathogenic bacteria adhesion. In fact, the composition of the microbiota is influenced by environmental factors such as diet, antibiotic therapy, and environmental exposure to microorganisms^26^.

Prebiotic foods are, by definition, non-digestible foods that specifically support the growth and/or activity of health-promoting bacteria that colonize the gastrointestinal tract. On the other hand, the role of omega-3 on microbiome composition and diversity has yet to be explored in human cohorts. Supplementation with DHA has been shown to help with oral and gastrointestinal diseases in which inflammation and bacterial dysbiosis play key roles^27^. Chronic low grade inflammation is often the result of an increase in plasma endotoxins, particularly lipopolysaccharides (LPS) derived from gut dysbiosis. The increase in plasma endotoxins leads to subsequent activation of the inflammasome and increased expression of inflammatory cytokines^24^. Analysis of gut microbiota and faecal transfer in mice has revealed that elevated tissue omega-3 fatty acids enhance intestinal production and secretion of intestinal alkaline phosphatase, which induces changes in the gut bacteria composition resulting in decreased lipopolysaccharide production and gut permeability, and ultimately, reduced metabolic endotoxemia and inflammation^24^.

A recent randomized, controlled clinical trial in an Indian population has shown that supplementation with omega 3 plus a probiotic has a greater beneficial effect on insulin sensitivity, lipid profile, and atherogenic index than the probiotic alone, although omega-3 supplementation showed only marginal effects on all the parameters^28^. A high omega-3 diet has also been shown to alter altered gut microbiota composition of drug-naïve patients with type 2 diabetes^29^. Such reports suggest an interaction between microbiome composition and intake of omega-3 fatty acids but a close examination of the links between omega-3 circulating levels and detailed microbiome composition in non-infant cohorts has not been explored to date.

Bacterial species, including those forming the human gut microbiome, are not well defined, and bacterial genomes are highly variable. Therefore regions used to identify bacteria vary in a continuum rather than clusters of similar sequences^30^. Bacteria that have 97% identity in a 16S rRNA gene variable region are considered to be the same taxa^31^. This is an arbitrary cut-off is thought to maximizes the grouping of bacteria classified as the same species while minimizing the grouping of bacteria classified as different species^32^. In order to determine how a batch of sequences should be partitioned into groups of 97% identity a clustering algorithm partitions the groups is used and taxonomic identity by matching the seed or central sequences with public databases are later assigned, the public database used was Greengenes in our case^33^. The resulting taxonomic groupings are known as Operational Taxonomic Units (OTUs), and are used consistently within the same experiment.

The aim of this study is to assess the association between omega-3 fatty acids serum levels and intake with microbiome composition diversity and with specific OTUs.

## Results

We analysed data from 876 female twins with 16 s microbiome data and circulating levels of fatty acids including DHA, total omega-3 fatty acids (FAW3), 18:2 linolenic acid (LNA), total omega-6 fatty acids (FAW6), total PUFA, and for comparison we also tested monounsaturated fatty acids; 16:1, 18:1 (MUFA), total saturated fatty acids (SFA), and total fatty acids (TotFA) measured at the same time point using the Brainshake NMR platform. Paired end reads covering the V4 region of the 16S rRNA gene were merged with a minimum overlap of 200nt using default parameters in the QIIME join_paired_ends.py script. Demultiplexed reads were then subject to chimera detection and removal on a per sample basis using *de novo* chimera detection in USEARCH, after which 290445606 reads were retained from a total of 317617494 reads across all TwinsUK samples. Samples with less than 10,000 reads were discarded. Within the subset of 1044 samples used in the present study the final read depth was 80865 ± 35718 (mean ± SD). 1044 samples used in the present study the final read depth was 80865 ± 35718 (mean ± SD).

The demographic characteristics of the study population are presented in Table 1.

**Table 1:** Descriptive characteristics of the 876 female twins studied, *mean*(*SD*).

	mean	SD
**Demographics**		
age, yrs	64.98	7.57
BMI, kg/m^2^	26.35	4.83
**Diversity measures**		
Shannon diversity	6.42	0.79
CHAO1	1976.97	667.21
Observed Species	876.52	255.98
Phylogenetic Diversity	67.99	19.15
Simpson diversity	0.95	0.04
**Serum Fatty Acids**		
DHA, mmol/l	0.14	0.05
FAW3, mmol/l	0.44	0.14
LNA, mmol/l	3.0	0.59
FAW6, mmol/l	3.58	0.64
PUFA, mmol/l	4.02	0.75
MUFA, mmol/l	2.63	0.62
SFA, mmol/l	3.42	0.69
TotFa, mmol/l	10.08	1.94
**Dietary intake**		
Fibre dietary intake, g/day	19.99	7.08
DHA dietary intake, g/day	0.35	0.86
EPA dietary intake, g/day	0.09	0.07

*DHA* docosahexaenoic acid, *FAW3* total omega-3, *LNA* the omega-6 linoleic acid 18:2, *FAW6* total omega 6 fatty acids, *PUFA* total polyunsaturated fatty acids, *MUFA* monounsaturated fatty acids; 16:1, 18:1, *SFA* saturated fatty acids, *TotFA* total fatty acids.

The serum circulating levels of the polyunsaturated fatty acids FAW3, FAW6, LNA, and DHA reflect in part the intake levels: we observe a significant correlation between dietary omega-3 fatty acid intake estimates from FFQs and serum levels of FAW3 (ρ = 0.168, p < 2.64 × 10^−7^).

### Omega-3 and omega-6 associate with microbiome diversity

**Figure 1:**
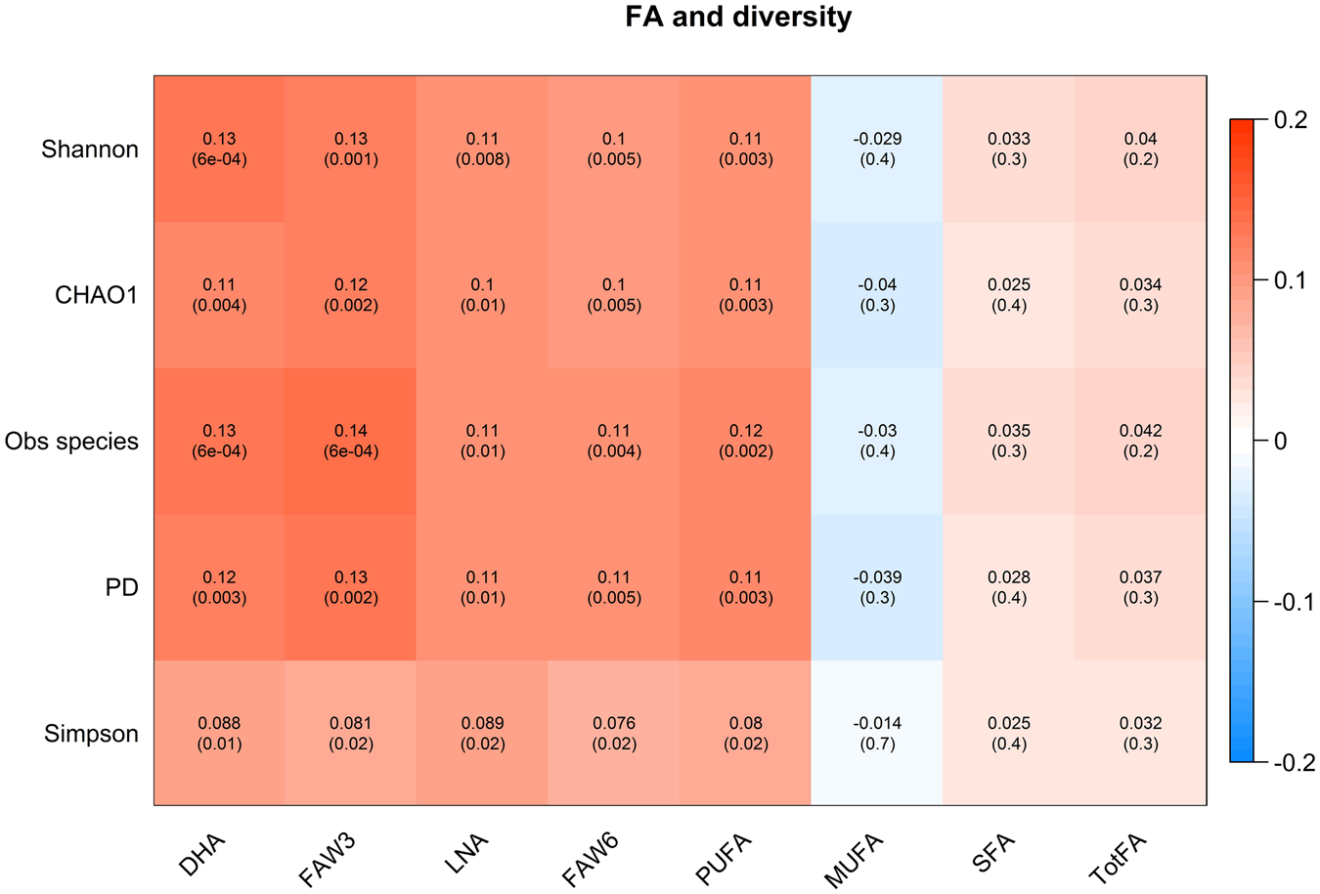
Each cell of the matrix contains the correlation between one serum fatty acid and a gut microbiome diversity measure and the corresponding p value. Analyses are adjusted for age, BMI and family relatedness. The table is color coded by correlation according to the table legend (*red* for positive and *blue* for negative correlations). *DHA* docosahexaenoic acid, *FAW3* total omega-3, *LNA* the omega-6 linoleic acid 18:2,*FAW6* total omega 6 fatty acids, *PUFA* total polyunsaturated fatty acids, *MUFA* monounsaturated fatty acids; 16:1, 18:1, *SFA* saturated fatty acids, *TotFA* total fatty acids.

After adjusting for age and BMI, we find that circulating PUFA levels measured as DHA, FAW3, LNA, FAW6, total PUFA are significantly and consistently associated with higher microbiome diversity across the 5 alpha diversity indexes that we computed: Shannon, Chao1, Simpson and phylogenetic diversity indices, as well as observed species (e.g., with Shannon’s diversity DHA: Beta(SE) = 0.13(0.04), P = 0.0006; FAW3: 0.13(0.04), P = 0.0011, LNA: 0.11(0.004), P = 0.008, FAW6: 0.10(0.04), P = 0.0047, total PUFA: 0.11(0.04), P = 0.003). The complete results are presented in Fig. 1. We found however no significant association between microbiome diversity and circulating levels of saturated fatty acids or monounsaturated fatty acids (Fig. 1).

The association between FAW6 and microbiome diversity became not significant when adjusted for either FAW3 or only DHA (Beta(SE) = 0.035(0.04), P = 0.39). Similarly after adjustment for FAW6, FAW3 (i.e. DHA + EPA) became not significant (Beta 0.070 (0.04), P = 0.09). On the other hand the association between microbiome alpha diversity and DHA only (excluding other omega-3 fatty acids) remained statistically significant adjusting for FAW6 levels (Beta 0.080 (0.036), P = 0.03). Therefore we decided to focus specifically on DHA levels. We tested for association between dietary intake of DHA and microbiome diversity and found a weaker association between DHA intake and microbiome diversity (Beta(SE) = 0.06(0.03), P = 0.0203). As dietary fibre intake positively correlates with the gut microbiome^34^, we also ran the analysis adjusting for fibre intake and the results remained consistent (Supplementary Table 2).

In our data we calculated an estimate of microbial beta-diversity using both weighted and unweighted UniFrac distances^30^ as implemented in QIIME^35^ and used principal coordinate analysis to examine its associations with omega-3 circulating levels, but did not find significant patterns [data not shown]. This is expected as DHA is not the main source of microbiome variation, rather it is only one of the many variables influencing it. However, the significant correlations of DHA and FAW3, respectively, with microbial alpha diversity do demonstrate an association of these metabolites with the overall microbiome composition. *DHA serum levels associate with OTU abundances*

We then investigated the association between OTUs and DHA and identified 38 OTUs significantly associated with serum levels of DHA after adjusting for covariates and multiple testing using FDR < 0.05 (Table 2). Out of 36 positive associations, 21 (58%) belong to the *Lachnospiraceaes*, 7 (19%) to the *Ruminococcacae* and 5 (14%) to the *Bacteroidetes*. Because some of these associations may simply reflect a correlation with microbiome diversity we further adjusted for Shannon’s index. We found that after adjustment for microbiome diversity the significant associations with individual OTUs (using a cut-off of FDR p < 0.05) remain nominally statistically significant. Therefore the associations with individual OTUs are not due exclusively to the correlation between *Lachnospiraceaes* abundance and measures of gut microbiome diversity.

**Table 2:** Associations between gut bacterial operational taxonomic units (OTUs) and serum levels of docosahexanoic acid (DHA).

Internal**	OTU*	BETA	SE	P	Q
otu1501	k__Bacteria; p__Firmicutes; c__Clostridia; o__Clostridiales; f__Lachnospiraceae; g__; s__	0.13	0.03	8.33 × 10^−7^	0.001
otu1453	k__Bacteria; p__Firmicutes; c__Clostridia; o__Clostridiales; f__Lachnospiraceae; g__; s__	0.13	0.03	9.11 × 10^−7^	0.001
otu1367	k__Bacteria; p__Firmicutes; c__Clostridia; o__Clostridiales; f__Lachnospiraceae; g__Lachnospira; s__	0.13	0.03	3.36 × 10^−6^	0.002
otu283	k__Bacteria; p__Firmicutes; c__Clostridia; o__Clostridiales; f__; g__; s__	0.10	0.02	2.61 × 10^−5^	0.009
otu1554	k__Bacteria; p__Firmicutes; c__Clostridia; o__Clostridiales; f__Lachnospiraceae	0.11	0.03	6.72 × 10^−5^	0.019
otu781	k__Bacteria; p__Firmicutes; c__Clostridia; o__Clostridiales; f__Lachnospiraceae; g__Lachnospira; s__	0.12	0.03	7.29 × 10^−5^	0.017
otu1355	k__Bacteria; p__Firmicutes; c__Clostridia; o__Clostridiales; f__Lachnospiraceae; g__; s__	0.11	0.03	7.76 × 10^−5^	0.016
otu845	k__Bacteria; p__Firmicutes; c__Clostridia; o__Clostridiales; f__; g__; s__	0.11	0.03	1.04 × 10^−4^	0.019
otu2057	k__Bacteria; p__Bacteroidetes; c__Bacteroidia; o__Bacteroidales; f__Bacteroidaceae; g__Bacteroides; s__	0.11	0.03	1.30 × 10^−4^	0.021
otu1793	k__Bacteria; p__Firmicutes; c__Erysipelotrichi; o__Erysipelotrichales; f__Erysipelotrichaceae; g__; s__	0.10	0.03	1.44 × 10^−4^	0.021
otu573	k__Bacteria; p__Firmicutes; c__Clostridia; o__Clostridiales; f__Lachnospiraceae; g__; s__	0.11	0.03	1.85 × 10^−4^	0.024
otu499	k__Bacteria; p__Firmicutes; c__Clostridia; o__Clostridiales; f__Lachnospiraceae; g__Coprococcus; s__	0.10	0.03	1.99 × 10^−4^	0.024
otu1933	k__Bacteria; p__Firmicutes; c__Clostridia; o__Clostridiales; f__Ruminococcaceae; g__Oscillospira; s__	0.10	0.03	2.32 × 10^−4^	0.026
otu1760	k__Bacteria; p__Firmicutes; c__Clostridia; o__Clostridiales; f__Lachnospiraceae; g__; s__	0.10	0.03	2.59 × 10^−4^	0.027
otu134	k__Bacteria; p__Firmicutes; c__Clostridia; o__Clostridiales; f__Ruminococcaceae; g__; s__	0.11	0.03	3.01 × 10^−4^	0.029
otu1061	k__Bacteria; p__Firmicutes; c__Clostridia; o__Clostridiales; f__Lachnospiraceae; g__; s__	0.09	0.03	3.20 × 10^−4^	0.029
otu1103	k__Bacteria; p__Firmicutes; c__Clostridia; o__Clostridiales; f__Lachnospiraceae; g__; s__	0.10	0.03	3.27 × 10^−4^	0.028
otu1212	k__Bacteria; p__Firmicutes; c__Clostridia; o__Clostridiales; f__Ruminococcaceae; g__Oscillospira; s__	0.10	0.03	3.69 × 10^−4^	0.030
otu867	k__Bacteria; p__Firmicutes; c__Clostridia; o__Clostridiales; f__Ruminococcaceae; g__; s__	0.11	0.03	3.83 × 10^−4^	0.029
otu1886	k__Bacteria; p__Bacteroidetes; c__Bacteroidia; o__Bacteroidales; f__Bacteroidaceae; g__Bacteroides; s__uniformis	0.09	0.03	3.85 × 10^−4^	0.028
otu769	k__Bacteria; p__Firmicutes; c__Clostridia; o__Clostridiales; f__Ruminococcaceae; g__; s__	0.10	0.03	4.21 × 10^−4^	0.029
otu1739	k__Bacteria; p__Firmicutes; c__Clostridia; o__Clostridiales; f__Lachnospiraceae	0.10	0.03	4.36 × 10^−4^	0.029
otu1074	k__Bacteria; p__Firmicutes; c__Clostridia; o__Clostridiales; f__Ruminococcaceae; g__Oscillospira; s__	0.10	0.03	4.37 × 10^−4^	0.027
otu169	k__Bacteria; p__Proteobacteria; c__Gammaproteobacteria; o__Enterobacteriales; f__Enterobacteriaceae	−0.09	0.03	5.80 × 10^−4^	0.035
otu533	k__Bacteria; p__Firmicutes; c__Clostridia; o__Clostridiales; f__Lachnospiraceae; g__; s__	0.10	0.03	6.56 × 10^−4^	0.038
otu271	k__Bacteria; p__Firmicutes; c__Clostridia; o__Clostridiales; f__Lachnospiraceae; g__Lachnospira; s__	0.10	0.03	6.58 × 10^−4^	0.036
otu2051	k__Bacteria; p__Firmicutes; c__Clostridia; o__Clostridiales; f__Lachnospiraceae; g__; s__	0.10	0.03	6.66 × 10^−4^	0.035
otu1672	k__Bacteria; p__Firmicutes; c__Clostridia; o__Clostridiales; f__Lachnospiraceae; g__Lachnospira; s__	0.10	0.03	6.73 × 10^−4^	0.035
otu2050	k__Bacteria; p__Bacteroidetes; c__Bacteroidia; o__Bacteroidales; f__Bacteroidaceae; g__Bacteroides; s__	0.09	0.03	7.84 × 10^−4^	0.039
otu436	k__Bacteria; p__Firmicutes; c__Clostridia; o__Clostridiales; f__Lachnospiraceae; g__; s__	0.10	0.03	9.21 × 10^−4^	0.044
otu1748	k__Bacteria; p__Bacteroidetes; c__Bacteroidia; o__Bacteroidales; f__Bacteroidaceae; g__Bacteroides; s__	0.09	0.03	9.22 × 10^−4^	0.043
otu2002	k__Bacteria; p__Firmicutes; c__Clostridia; o__Clostridiales; f__Lachnospiraceae; g__; s__	0.09	0.03	9.94 × 10^−4^	0.045
otu205	k__Bacteria; p__Firmicutes; c__Clostridia; o__Clostridiales; f__Lachnospiraceae; g__Lachnospira; s__	0.10	0.03	9.99 × 10^−4^	0.044
otu1063	k__Bacteria; p__Proteobacteria; c__Gammaproteobacteria; o__Enterobacteriales; f__Enterobacteriaceae; g__; s__	−0.09	0.03	1.06 × 10^−3^	0.045
otu55	k__Bacteria; p__Firmicutes; c__Clostridia; o__Clostridiales; f__Lachnospiraceae; g__Roseburia; s__	0.10	0.03	1.06 × 10^−3^	0.044
otu662	k__Bacteria; p__Firmicutes; c__Clostridia; o__Clostridiales; f__Lachnospiraceae; g__; s__	0.10	0.03	1.10 × 10^−3^	0.044
otu1621	k__Bacteria; p__Firmicutes; c__Clostridia; o__Clostridiales; f__Ruminococcaceae; g__; s__	0.10	0.03	1.15 × 10^−3^	0.045
otu2006	k__Bacteria; p__Bacteroidetes; c__Bacteroidia; o__Bacteroidales; f__Bacteroidaceae; g__Bacteroides; s__uniformis	0.09	0.03	1.29 × 10^−3^	0.049

Associations are expressed as the regression coefficient and standard error (adjusted for age, body mass index, family relatedness). P-values and FDR (Q-values) are shown. ^*^OTUs are only analytical units which could represent individual strains or species, as such more than one can be assigned to the same taxonomy. **The internal name is meaningless and just randomly generated but it is there to indicate that there are separate hits.

We also explored whether the effects of percent DHA differed from those of absolute concentrations. We find that the same associations hold when absolute concentrations of DHA and percentage of DHA of all other fatty acids are seen both with regards to microbiome diversity (Shannon index association DHA% Shannon: Beta 0.15(0.04), P = 0.001 DHA absolute concentration 0.13(0.04), P = 0.001) and with regards to the most highly associated OTU (otu1505 association DHA% Shannon: Beta 0.11(0 .03), P = 3.0 × 10^−5^ vs DHA absolute concentration Beta 0.13(SE 0.03) P = 8.33 × 10^−7^).

### Faecal metabolites associated with DHA levels and OTU abundances

To further understand the link between DHA levels and the gut microbiome we assessed the correlation between DHA circulating levels and faecal metabolites measured using commercial metabolomic panel (Metabolon Inc, Supplementary Table 3) in a subset of 707 individuals with data available. After adjusting for multiple testing using FDR < 0.05, the faecal metabolites associated with DHA serum levels were faecal levels of the omega-3 fatty acids EPA (Beta(SE) = 0.15(0.037), P = 4.35 × 10^−5^, FDR = 0.01), N-carbamylglutamate (0.15(0.039), P = 1.21 × 10^−4^, FDR = 0.02) and the dipeptide anserine (0.13(0.036), P = 5.3 × 10^−4^, FDR = 0.04) commonly found in fish and poultry meat^36^ and hence is likely to be positively correlated with fish intake) (Supplementary Table 1). We hypothesized that the association between some of the OTUs and DHA may be mediated by the levels in the gut of N-carbamylglutamate (NCG). We found that several OTUs whose abundance is associated with DHA serum levels are also associated with NCG faecal levels (Table 3). The association remains significant after adjustment for DHA, however some of the DHA associations with these OTUs are attenuated when adjusting for NCG levels (Table 3) suggesting that the correlation between the abundances some of these OTUs and DHA serum levels could be mediated by NCG faecal concentrations.

**Table 3:** Association between gut bacterial operational taxonomic units (OTUs) and N-carbamylglutamate (NCG) in faeces unadjusted (unadj) and adjusting for DHA serum levels (adj).

Internal	OTU		BETA	NCG	P	BETA	DHA	P
				SE			SE	
otu1621	p__Firmicutes; c__Clostridia; o__Clostridiales; f__Ruminococcaceae; g__; s__	Unadj	0.15	0.04	9.71 × 10^−4^	0.10	0.03	0.001
		Adj	0.14	0.05	2.81 × 10^−3^	0.09	0.04	0.02
otu573	p__Firmicutes; c__Clostridia; o__Clostridiales; f__Lachnospiraceae; g__; s__	Unadj	0.13	0.04	1.15 × 10^−3^	0.11	0.03	1.85 × 10^−4^
		Adj	0.12	0.04	3.79 × 10^−3^	0.08	0.03	0.02
**otu769**	**p__Firmicutes; c__Clostridia; o__Clostridiales; f__Ruminococcaceae; g__; s__**	**Unadj**	**0.12**	**0.04**	**2.92 × 10** ^**−3**^	**0.10**	**0.03**	**4.21 × 10** ^**−4**^
		**Adj**	**0.11**	**0.04**	**0.01**	**0.07**	**0.03**	**0.02**
otu1793	p__Firmicutes; c__Erysipelotrichi; o__Erysipelotrichales; f__Erysipelotrichaceae; g__; s__	Unadj	0.11	0.04	8.89 × 10^−3^	0.10	0.03	1.44 × 10^−4^
		Adj	0.1	0.04	0.02	0.10	0.03	1.44 × 10^−4^
otu436	p__Firmicutes; c__Clostridia; o__Clostridiales; f__Lachnospiraceae; g__; s__	Unadj	0.1	0.04	0.012	0.10	0.03	9.21 × 10^−4^
		Adj	0.09	0.04	0.03	0.08	0.04	0.02
otu2050	p__Bacteroidetes; c__Bacteroidia; o__Bacteroidales; f__Bacteroidaceae; g__Bacteroides; s__	Unadj	0.1	0.04	0.013	0.09	0.03	7.84 × 10^−4^
		Adj	0.08	0.04	0.03	0.09	0.03	7.84 × 10^−4^
otu1886	p__Bacteroidetes; c__Bacteroidia; o__Bacteroidales; f__Bacteroidaceae; g__Bacteroides; s__uniformis	Unadj	0.09	0.04	0.015	0.09	0.03	3.85 × 10^−4^
		Adj	0.08	0.04	0.02	0.09	0.03	0.01
**otu1453**	**p__Firmicutes; c__Clostridia; o__Clostridiales; f__Lachnospiraceae; g__; s__**	**Unadj**	**0.09**	**0.04**	**0.023**	**0.13**	**0.03**	**9.11 × 10** ^**−7**^
		**Adj**	**0.08**	**0.04**	***0.06***	**0.10**	**0.03**	**0.0008**
**otu55**	**p__Firmicutes; c__Clostridia; o__Clostridiales; f__Lachnospiraceae; g__Roseburia; s__**	**Unadj**	**0.09**	**0.04**	**0.03**	**0.10**	**0.03**	**1.06 × 10** ^**−3**^
		**Adj**	**0.08**	**0.04**	***0.06***	**0.08**	**0.04**	**0.02**
otu1061	p__Firmicutes; c__Clostridia; o__Clostridiales; f__Lachnospiraceae; g__; s__	Unadj	0.09	0.04	0.03	0.09	0.03	3.20 × 10^−4^
		Adj	0.08	0.04	0.06	0.08	0.03	0.01
otu134	p__Firmicutes; c__Clostridia; o__Clostridiales; f__Ruminococcaceae; g__; s__	Unadj	0.09	0.04	0.032	0.11	0.03	3.01 × 10^−4^
		Adj	0.08	0.04	0.07	0.10	0.03	0.001
otu1074	p__Firmicutes; c__Clostridia; o__Clostridiales; f__Ruminococcaceae; g__Oscillospira; s__	Unadj	0.08	0.04	0.042	0.10	0.03	4.37 × 10^−4^
		Adj	0.06	0.04	0.09	0.07	0.03	0.04
**otu499**	**p__Firmicutes; c__Clostridia; o__Clostridiales; f__Lachnospiraceae; g__Coprococcus; s__**	**Unadj**	**0.09**	**0.04**	**0.043**	**0.10**	**0.03**	**1.99 × 10** ^**−4**^
		**Adj**	**0.08**	**0.04**	**0.07**	**0.06**	**0.03**	**0.06**
otu1748	p__Bacteroidetes; c__Bacteroidia; o__Bacteroidales; f__Bacteroidaceae; g__Bacteroides; s__	Unadj	0.08	0.04	0.044	0.09	0.03	9.22 × 10^−4^
		Adj	0.06	0.04	0.1	0.10	0.03	0
otu2051	p__Firmicutes; c__Clostridia; o__Clostridiales; f__Lachnospiraceae; g__; s__	Unadj	0.08	0.04	0.046	**0.10**	**0.03**	**6.66 × 10** ^**−4**^
		Adj	0.07	0.04	0.09	0.09	0.03	0.01

The association of the same OTUs with DHA adjusted and unadjusted for NCG is also shown. All analyses are adjusted for age, BMI, and family relatedness.

## Discussion

In this study we show in a population based cohort of middle aged and elderly women that circulating levels of omega-3 fatty acids are associated with higher microbiome diversity and with a higher abundance of OTUs belonging to the *Lachnospiraceae* family.

Omega-3 levels in humans are determined by dietary intake and by conversion of alpha-linolenic acid (ALA) to DHA. Although it is possible that the gut microbiome affects absorption of these fatty acids, given that most of the absorption of fatty acids takes place in the small intestine, it seems more likely that the link that we observed between DHA and microbiome is mediated by circulating DHA, or by DHA incorporated into the large intestines or by other intermediates that DHA affects (e.g. D-series resolvins).

Having tested five different ecological measures of microbiome diversity we find that all of them are positively correlated with higher serum levels of omega-3 and omega-6 fatty acids and all of them show the same pattern with regards to the various fatty acid measures tested. Higher gut microbiome diversity is linked to lower inflammation^37, 38^, thus our data reinforce the notion that omega-3 fatty acids are linked to lower gut inflammation.

We also identified 38 OTUs associated with circulating levels of DHA. In particular we find that positive associations were enriched for the *Lachnospiraceae* family*. Lachnospiraceae* are one of the main taxonomic groups of the human gut where they function to degrade complex polysaccharides to short-chain fatty acids (SCFAs) such as acetate, butyrate, and propionate that are used by the host for energy^39^. SCFAs are the end products of fermentation of dietary fibres by the anaerobic intestinal microbiota and have been shown to exert multiple beneficial effects on mammalian energy metabolism. The mechanisms underlying these effects encompass the complex interplay between diet, gut microbiota, and host energy metabolism^40^. Members of the *Lachnospiraceae* family are found in higher abundance in herbivorous animals^41^. The wide range of functions carried out by *Lachnospiraceae* may influence their relative abundance in gut communities of different hosts. In humans, members of this family have been associated with protection against *C. difficile* infections^42^ and obesity^43^. They are also known as potent short-chain fatty acid producers^44^, On the other hand, we also find members of the *Ruminococcaceae* associated with increased levels of DHA and subtypes of some of this family have been implicated in obesity^45^.

The anti-inflammatory benefits of omega-3 PUFAs on gut microbiome composition may be attributed to the products of DHA metabolism, in particular those resulting from endogenous lipoxygenase-catalyzed hydroxylation of DHA, which in turn produces resolvins and protectin D1 through acetylation of the cyclooxygenase-2 enzyme^46^. Numerous reports describe protective effects of EPA- and DHA-derived mediators in experimental models of inflammatory bowel diseases^47, 48^ (reviewed in ref. 49). There is also some evidence of some benefit from supplementation of omega-3 fatty acids in humans affected by inflammatory bowel conditions^50–52^.

Modulation of these inflammatory pathways may similarly explain how DHA could reduce bowel inflammatory levels in bowel conditions where the ability of epithelial and immune cells in the intestine to differentiate between pathogenic and commensal bacteria leads to prolonged activation of nuclear factor-κB^27^. NF κB is a pro-inflammatory transcription factor which triggers overproduction of inflammatory cytokines. Inflammation of the gastrointestinal tract in turn interrupts the natural balance between the mucosal immune system and normal gut microbiota^53^. Although our data indicate that the DHA effect is independent of fibre intake, it is well known that SCFAs result from microbial fermentation of fibre^40^. Interventional nutritional studies may be required to quantify and dissect the contribution of these two types of dietary components on microbiome composition and SCFA production.

Moreover, most of the bacterial grouping that we find associated with increased levels of serum DHA are also negatively correlated with Crohn’s disease severity and intestinal inflammation (the Pediatric Crohn’s Disease Activity Index) such as *Lachnospiraceae*, *Coprococcus*, *Roseburia, Ruminococcus*, and *Clostridium* ^54^. These bacteria are known to be major producers of the SCFA butyrate^55, 56^.

In addition, we report that DHA serum levels correlate with the faecal concentration of NCG after adjusting for multiple testing. This carbamylated aminoacid, is available as a synthetic compound, but may also be generated in nature by protein carbamylation^57^. Given that the other faecal compounds strongly associated with DHA serum levels are omega-3 faecal levels and a dipeptide considered as a marker of animal protein (such as fish) intake, the abundance of this compound in the faeces is unlikely to be the result of intake of a supplement containing it. NCG is a precursor of arginine, a structural analogue of N- acetylglutamate and selective activator of the first enzyme of the urea cycle^58^. In animal studies NCG supplementation has been shown to improve arginine synthesis in enterocytes^59^, to regulate signalling pathways (such as signal transduction and activator of transcription 3 (Stat3), protein kinase B (PKB), and 70-kDa ribosomal protein S6 kinase)^59^ and to enhance intestinal growth as well as heat shock protein-70 expression^60^. Importantly, it also reduces oxidative stress in the gut^61, 62^ and alters intestinal gene expression^63^. Some of the NCG effects may be mediated via arginine which helps maintain intestinal homeostasis, preserves the integrity of the intestinal epithelium under stress, and prevents intestinal permeability and bacterial translocation^64, 65^.

Given the beneficial effects of NCG in the mammalian gut, part of the explanation for the association between DHA and gut microbiome composition might be that the presence of DHA favours the production of NCG by the gut microbiota. This in turn is likely to result in improved gut function and reduced oxidative stress. We see that the association between DHA circulating levels and some of the OTUs (such as *Coprococcus*, *Oscillospira*, *Roseburia*) is attenuated when we adjusted for NCG faecal levels (Table 3) suggesting that some of the DHA effect may be mediated by NCG. These are some of the bacteria that, as mentioned above, have been linked to either reduced intestinal inflammation or reduced risk of Crohn’s disease and are butyrate producers.

Although these results are only observational and cross-sectional they raise the possibility that omega-3 fatty acids may represent an important dietary supplement also to improve gut microbiome health. Our data suggest that the effect of omega-3 FA, in particular DHA, is independent of fibre intake. These data also support the hypothesis that some of the reported beneficial effects of omega-3 supplementation may be due to their effect on the gut microbiome. Nonetheless, intake of omega-3 fatty acids tends to correlate with a healthier lifestyle in general^1^ and therefore some of the effects of DHA on the gut may be indirect. This cannot be directly established in our study given the cross-sectional nature of the data analysed.

We note several study limitations. First, given the cross-sectional nature of the data, we cannot establish whether it is omega-3 affecting microbiome diversity or the other way round. On the other hand, it is well known that omega-3 circulating levels are reflective of dietary intake^66^ and although the associations we find with estimates of omega-3 dietary intake are weaker than with serum levels, this is likely to simply reflect the error of accurate estimates of intake from food questionnaire data compared to the accuracy of serum level measurements. Therefore, we hypothesize that it is intake of PUFAs and in particular of DHA that results in higher microbiome diversity and increased *Lachnospiraceaes* abundance. Our data are consistent with a recent randomized crossover clinical study in obese individuals where the effects of the gut microbiome were compared between supplementation with high oleic acid canola oil, and canola oil plus DHA. A principal analysis component carried by the authors revealed a strong enrichment of *Lachnospiraceae* in the canola plus DHA vs canola oil group, and more modest enrichments for *Coprococcus* and *Ruminococcaceae* ^67^. Second, our study also lacked direct measurement of SCFAs in order to show a direct correlation between DHA serum levels and SCFAs in the gut. Third, our cohort sonsists exclusively of middle aged and elderly women of European descent. The lack of male participants in our in this study limits the generalisability of our results since gender differences in the gut microbiome have been reported in humans^68^. Moreover, estrogens cause higher DHA concentrations in women than in men, probably by upregulating synthesis of DHA from alpha linoleic acid^69^. Additional studies including men may show stronger or weaker associations with omega-3 fatty acids. Fourth, we have used FFQs rather than other methods for assessing nutrient intake. It has been argued that 7-day diet diaries or records add useful information above and beyond FFQ remains and have higher reproducibility and lower error rates^70^. However,, the value of FFQs for assessing dietary composition has been documented objectively by correlations with biochemical indicators and the prediction of outcomes in prospective studies^71^. Moreover, the key results we report are with serum levels of DHA, which are highly correlated with intake figures estimated from FFQs.

We also note several study strengths. Our study has the largest sample size studied to date with regards to the relationship between omega-3 fatty acids and the gut microbiome composition. The study was able to compare various NMR measures of omega-3, omega-6 in addition to dietary intake of omega-3 fatty acids. The study is one of the very few to investigate the link between microbiome associations and the faecal metabolome.

In conclusion, our data indicate a strong correlation between omega-3 fatty acids and microbiome composition and suggest that supplementation with PUFAs may be considered along with prebiotic and probiotic supplementation aimed at improving the microbiome composition and diversity. The study also suggests the translational potential NCG as a supplement to improve gut function and microbiome composition.

## Methods

### Study population

Study subjects were female twins enrolled in the TwinsUK registry, a national register of adult twins recruited as volunteers without selecting for any particular disease or trait traits^72^. In this study, we analysed data from 876 female twins with 16 s microbiome data and serum DHA, total omega-3, omega-6 and LA acids measured at the same time using the Brainshake NMR platform. The study was approved by NRES Committee London–Westminster, all experiments were performed in accordance with relevant guidelines and regulations, and all twins provided informed written consent.

#### NMR Metabolomics

circulating levels of DHA, FWA3, LNA, FAW6, total PUFA, total MUFA, total SFA, and TotFA) were measured by Brainshake Ltd, Finland, (https://www.brainshake.fi/) from fasting serum samples using 500 Mhz and 600 Mhz proton nuclear magnetic resonance spectroscopy as previously described^73^. Traits were log-transformed and then scaled to standard deviation units, as previously proposed by Würtz *et al*.^3^. For the metabolites containing zeroes, 1 was added to all values of that metabolite before log-transformation.

#### Fibre and fatty acid intake

A validated 131-item semi-quantitative Food Frequency Questionnaire (FFQ) established for the EPIC (European Prospective Investigations into Cancer and Nutrition)-Norfolk study^74^ was used to assess dietary intake. Estimated intakes of essential fatty acids and fibre (in grams per day) were derived from the UK Nutrient Database^75^ and were adjusted for energy intake using the residual method prior to analysis^71^.

#### Microbiota analysis

The stool DNA extraction is detailed in Goodrich *et al*.^76^ of 100 mg were taken from the sample and used for extraction. There was no homogenisation prior to this step. Faecal samples were collected and the composition of the gut microbiome was determined by 16 S rRNA gene sequencing carried out as previously described^77, 78^. Briefly, the V4 region of the 16 S rRNA gene was amplified and sequenced on Illumina MiSeq. Reads were then summarised to OTUs using open reference clustering Greengenes v13_8 at 97% sequence similarity^78^. OTU counts were converted to log transformed relative abundances, with zero counts handled by the addition of an arbitrary value (10^−6^). The residuals of the OTU abundances were taken from linear models, accounting for technical covariates including sequencing depth, sequencing run, sequencing technician and sample collection method. These residuals were inverse normalised, to make them normally distributed, and used in downstream parametric analyses. This approach allows us to adjust for potential confounders using parametric methods and is justified by both the sample size available and the normal distribution of the transformed OTU abundances.

The OTU table was rarefied to a depth of 10 000 OTUs per sample and five measures of gut microbiome alpha diversity were computed: Shannon, Chao1, Simpson and phylogenetic diversity indices, as well as observed species. Alpha diversity indexes were standardised to have mean 0 and SD 1.

### Faecal metabolomics

Metabolite concentrations were measured from 707 faecal samples by Metabolon Inc., Durham, US, using an untargeted LC/MS platform as previously describe^79, 80^. Here we analysed 424 metabolites of known chemical identity observed in at least 80% of all samples. Metabolites were scaled by run-day medians and inverse normalised as the metabolite concentrations were not normally distributed. We imputed the missing values using the minimum run day measures.

### Statistical analysis

We assessed the association between circulating serum levels of DHA, FAW3, LNA, FAW6, total PUFA, total MUFA, total SFA, TotFA by using random intercept linear regression adjusting for age, BMI and family relatedness. Linear regression was also employed to investigate the association between OTUs and DHA adjusting for covariates and multiple testing using false discovery rate (FDR < 0.05). We then calculated the principal coordinates from both weighted and unweighted UniFrac distances, a measure of microbial beta-diversity^81^, as implemented in QIIME^35^ to further examine the associations between omega-3 circulating levels and microbiome composition.

Finally, we assessed the correlation between DHA circulating levels and faecal metabolites in a sub-analysis of 707 individuals using linear regression adjusting for age, BMI, family relatedness and multiple testing (FDR < 0.05). We then tested the associations between the significant faecal metabolites and the previously identified OTU adjusting also for DHA.

## Electronic supplementary material

Supplementary Tables
