# Supplementary material for: Omega-3 fatty acids correlate with gut microbiome diversity and production of N-carbamylglutamate in middle aged and elderly women
Source: Sci Rep. 2017 Sep 11;7:11079. doi: 10.1038/s41598-017-10382-2 (PMC5593975; doi:10.1038/s41598-017-10382-2)
Supplement: Supplementary file 1 — Supplementary Tables [file 41598_2017_10382_MOESM1_ESM.pdf]

# Omega-3 fatty acids correlate with gut microbiome diversity and production of N-carbamylglutamate in middle aged and elderly women

Cristina Menni[a], Jonas Zierer[a], Tess Pallister[a], Matthew A Jackson[a], Tao Long[b], Robert P Mohney[c], Claire J Steves[a], Tim D Spector[a], Ana M Valdes[a,d,e]

[a] Department of Twin Research and Genetic Epidemiology, Kings College London, London, UK.

[b] Sanford Burnham Prebys, USA

[c]Metabolon Inc., Raleigh-Durham, NC 27709, USA.

[d]School of Medicine , Nottingham City Hospital, Hucknall Road, Nottingham, UK.

[e] NIHR Nottingham Biomedical Research Centre, Nottingham, UK.

**Corresponding author:** Dr Ana M Valdes

School of Medicine Clinical Sciences Building, Nottingham City Hospital, Hucknall Road, Nottingham, NG5 1PB, UK

Phone number: +44 (0)115 823 1954; Fax number:+44(0) 115 823 1757

email: Ana.Valdes@nottingham.ac.uk

**Supplementary Table 1. List of faecal metabolites significantly associated to DHA circulating levels (FDR<0.05)adjusting for age, BMI and family relatedness.**

| Super Pathway | Sub pathway | Metabolite | BETA | SE | P | Q |
|---|---|---|---|---|---|---|
| **Lipid** | Polyunsaturated Fatty Acid (n3 and n6) | eicosapentaenoate (EPA; 20:5n3) | 0.15 | 0.04 | $4.35 \times 10^{-5}$ | 0.01 |
| **Xenobiotics** | Carbamylated aminoacid | N-carbamylglutamate | 0.15 | 0.04 | $1.21 \times 10^{-4}$ | 0.02 |
| **Peptide** | Dipeptide Derivative | anserine | 0.13 | 0.04 | $5.39 \times 10^{-4}$ | 0.04 |

**Supplementary Table 2. Associations between five measures of microbiome diversity and circulating levels of PUFA adjusting for age, BMI fibre intake and family relatedness**

| | SHANNON DIVERSITY | | | CHAO1 | | | OBSERVED SPECIES | | | Phylogenetic Diversity | | | SIMPSON | | |
|---|---|---|---|---|---|---|---|---|---|---|---|---|---|---|---|
| | **Beta** | **SE** | **P** | **Beta** | **SE** | **P** | **Beta** | **SE** | **P** | **Beta** | **SE** | **P** | **Beta** | **SE** | **P** |
| **DHA** | 0.13 | 0.04 | 0.002 | 0.12 | 0.04 | 0.003 | 0.14 | 0.04 | 0.001 | 0.12 | 0.04 | 0.01 | 0.09 | 0.04 | 0.02 |
| **FAW3** | 0.12 | 0.04 | 0.005 | 0.13 | 0.04 | 0.003 | 0.13 | 0.04 | 0.002 | 0.12 | 0.05 | 0.01 | 0.08 | 0.04 | 0.05 |
| **LA (18:2)** | 0.10 | 0.05 | 0.02 | 0.10 | 0.05 | 0.03 | 0.10 | 0.05 | 0.03 | 0.10 | 0.05 | 0.03 | 0.09 | 0.04 | 0.04 |
| **FAW6** | 0.09 | 0.04 | 0.02 | 0.10 | 0.04 | 0.01 | 0.10 | 0.04 | 0.01 | 0.10 | 0.04 | 0.02 | 0.08 | 0.04 | 0.05 |

DHA=22:6 docosahexaenoic acid;, FAW3= Omega-3 fatty acids; 18:2, LA= linoleic acid;; FAW6=omega-6 fatty acids

**Supplementary Table 3. List of faecal metabolites measured using commercial metabolomic panel Metabolon Inc**

| Super-pathway | Sub-pathway | Metabolite name |
|---|---|---|
| **Amino Acid** | Alanine and Aspartate Metabolism | 3-sulfo-L-alanine |
| **Amino Acid** | Alanine and Aspartate Metabolism | alanine |
| **Amino Acid** | Alanine and Aspartate Metabolism | asparagine |
| **Amino Acid** | Alanine and Aspartate Metabolism | aspartate |
| **Amino Acid** | Alanine and Aspartate Metabolism | N-acetylalanine |
| **Amino Acid** | Alanine and Aspartate Metabolism | N-acetylasparagine |
| **Amino Acid** | Alanine and Aspartate Metabolism | N-acetylaspartate (NAA) |
| **Amino Acid** | Alanine and Aspartate Metabolism | N-carbamoylalanine |
| **Amino Acid** | Alanine and Aspartate Metabolism | N-methylalanine |
| **Amino Acid** | Creatine Metabolism | creatine |
| **Amino Acid** | Creatine Metabolism | creatinine |
| **Amino Acid** | Creatine Metabolism | N-carbamoylsarcosine |
| **Amino Acid** | Glutamate Metabolism | carboxyethyl-GABA |
| **Amino Acid** | Glutamate Metabolism | gamma-aminobutyrate (GABA) |
| **Amino Acid** | Glutamate Metabolism | glutamate |
| **Amino Acid** | Glutamate Metabolism | glutamate, gamma-methyl ester |
| **Amino Acid** | Glutamate Metabolism | glutamine |
| **Amino Acid** | Glutamate Metabolism | N-acetylglutamate |
| **Amino Acid** | Glutamate Metabolism | N-acetylglutamine |
| **Amino Acid** | Glutamate Metabolism | N-methylglutamate |
| **Amino Acid** | Glutamate Metabolism | pyroglutamine* |
| **Amino Acid** | Glutamate Metabolism | S-1-pyrroline-5-carboxylate |
| **Amino Acid** | Glutathione Metabolism | 5-oxoproline |
| **Amino Acid** | Glycine, Serine and Threonine Metabolism | allo-threonine |
| **Amino Acid** | Glycine, Serine and Threonine Metabolism | betaine |
| **Amino Acid** | Glycine, Serine and Threonine Metabolism | dimethylglycine |
| **Amino Acid** | Glycine, Serine and Threonine Metabolism | glycine |
| **Amino Acid** | Glycine, Serine and Threonine Metabolism | N-acetylglycine |
| **Amino Acid** | Glycine, Serine and Threonine Metabolism | N-acetylserine |
| **Amino Acid** | Glycine, Serine and Threonine Metabolism | N-acetylthreonine |
| **Amino Acid** | Glycine, Serine and Threonine Metabolism | O-acetylhomoserine |
| **Amino Acid** | Glycine, Serine and Threonine Metabolism | serine |
| **Amino Acid** | Glycine, Serine and Threonine Metabolism | threonine |
| **Amino Acid** | Guanidino and Acetamido Metabolism | 1-methylguanidine |
| **Amino Acid** | Guanidino and Acetamido Metabolism | 4-guanidinobutanoate |
| **Amino Acid** | Histidine Metabolism | 1-methylimidazoleacetate |
| **Amino Acid** | Histidine Metabolism | 3-methylhistidine |
| **Amino Acid** | Histidine Metabolism | 4-imidazoleacetate |
| **Amino Acid** | Histidine Metabolism | cis-urocanate |
| **Amino Acid** | Histidine Metabolism | formiminoglutamate |
| **Amino Acid** | Histidine Metabolism | histidine |
| **Amino Acid** | Histidine Metabolism | imidazole lactate |

| | | |
|---|---|---|
| **Amino Acid** | Histidine Metabolism | imidazole propionate |
| **Amino Acid** | Histidine Metabolism | N-acetylhistidine |
| **Amino Acid** | Histidine Metabolism | trans-urocanate |
| **Amino Acid** | Leucine, Isoleucine and Valine Metabolism | 2,3-dimethylsuccinate |
| **Amino Acid** | Leucine, Isoleucine and Valine Metabolism | 2-hydroxy-3-methylvalerate |
| **Amino Acid** | Leucine, Isoleucine and Valine Metabolism | 3-methyl-2-oxobutyrate |
| **Amino Acid** | Leucine, Isoleucine and Valine Metabolism | 3-methyl-2-oxovalerate |
| **Amino Acid** | Leucine, Isoleucine and Valine Metabolism | 4-methyl-2-oxopentanoate |
| **Amino Acid** | Leucine, Isoleucine and Valine Metabolism | alpha-hydroxyisocaproate |
| **Amino Acid** | Leucine, Isoleucine and Valine Metabolism | alpha-hydroxyisovalerate |
| **Amino Acid** | Leucine, Isoleucine and Valine Metabolism | ethylmalonate |
| **Amino Acid** | Leucine, Isoleucine and Valine Metabolism | isoleucine |
| **Amino Acid** | Leucine, Isoleucine and Valine Metabolism | isovalerate (C5) |
| **Amino Acid** | Leucine, Isoleucine and Valine Metabolism | isovalerylglycine |
| **Amino Acid** | Leucine, Isoleucine and Valine Metabolism | leucine |
| **Amino Acid** | Leucine, Isoleucine and Valine Metabolism | methylsuccinate |
| **Amino Acid** | Leucine, Isoleucine and Valine Metabolism | N-acetylisoleucine |
| **Amino Acid** | Leucine, Isoleucine and Valine Metabolism | N-acetylleucine |
| **Amino Acid** | Leucine, Isoleucine and Valine Metabolism | N-acetylvaline |
| **Amino Acid** | Leucine, Isoleucine and Valine Metabolism | valine |
| **Amino Acid** | Lysine Metabolism | 2-aminoadipate |
| **Amino Acid** | Lysine Metabolism | 5-aminovalerate |
| **Amino Acid** | Lysine Metabolism | 6-oxopiperidine-2-carboxylic acid |
| **Amino Acid** | Lysine Metabolism | glutarate (pentanedioate) |
| **Amino Acid** | Lysine Metabolism | lysine |
| **Amino Acid** | Lysine Metabolism | N2,N6-diacetyllysine |
| **Amino Acid** | Lysine Metabolism | N2-acetyllysine |
| **Amino Acid** | Lysine Metabolism | N6,N6,N6-trimethyllysine |
| **Amino Acid** | Lysine Metabolism | N6-acetyllysine |
| **Amino Acid** | Lysine Metabolism | N6-carboxyethyllysine |
| **Amino Acid** | Lysine Metabolism | N6-formyllysine |
| **Amino Acid** | Lysine Metabolism | N-acetyl-cadaverine |
| **Amino Acid** | Lysine Metabolism | pipecolate |
| **Amino Acid** | Lysine Metabolism | saccharopine |
| **Amino Acid** | Methionine, Cysteine, SAM and Taurine Metabolism | 2-aminobutyrate |
| **Amino Acid** | Methionine, Cysteine, SAM and Taurine Metabolism | 2-hydroxybutyrate/2-hydroxyisobutyrate |
| **Amino Acid** | Methionine, Cysteine, SAM and Taurine Metabolism | cysteine |
| **Amino Acid** | Methionine, Cysteine, SAM and Taurine Metabolism | cysteine s-sulfate |
| **Amino Acid** | Methionine, Cysteine, SAM and Taurine Metabolism | cysteine sulfinic acid |
| **Amino Acid** | Methionine, Cysteine, SAM and Taurine Metabolism | cystine |
| **Amino Acid** | Methionine, Cysteine, SAM and Taurine Metabolism | methionine |
| **Amino Acid** | Methionine, Cysteine, SAM and Taurine | methionine sulfoxide |

| | | |
|---|---|---|
| | Metabolism | |
| **Amino Acid** | Methionine, Cysteine, SAM and Taurine Metabolism | N-acetylcysteine |
| **Amino Acid** | Methionine, Cysteine, SAM and Taurine Metabolism | N-acetylmethionine |
| **Amino Acid** | Methionine, Cysteine, SAM and Taurine Metabolism | N-acetylmethionine sulfoxide |
| **Amino Acid** | Methionine, Cysteine, SAM and Taurine Metabolism | N-formylmethionine |
| **Amino Acid** | Methionine, Cysteine, SAM and Taurine Metabolism | taurine |
| **Amino Acid** | Phenylalanine and Tyrosine Metabolism | (R)-salsolinol |
| **Amino Acid** | Phenylalanine and Tyrosine Metabolism | 3-(3-hydroxyphenyl)propionate |
| **Amino Acid** | Phenylalanine and Tyrosine Metabolism | 3-(4-hydroxyphenyl)lactate (HPLA) |
| **Amino Acid** | Phenylalanine and Tyrosine Metabolism | 3-(4-hydroxyphenyl)propionate |
| **Amino Acid** | Phenylalanine and Tyrosine Metabolism | 3-phenylpropionate (hydrocinnamate) |
| **Amino Acid** | Phenylalanine and Tyrosine Metabolism | 4-hydroxycinnamate |
| **Amino Acid** | Phenylalanine and Tyrosine Metabolism | 4-hydroxyphenylacetate |
| **Amino Acid** | Phenylalanine and Tyrosine Metabolism | 4-hydroxyphenylpyruvate |
| **Amino Acid** | Phenylalanine and Tyrosine Metabolism | cis-4-hydroxycyclohexylacetic acid |
| **Amino Acid** | Phenylalanine and Tyrosine Metabolism | dihydrocaffeate |
| **Amino Acid** | Phenylalanine and Tyrosine Metabolism | N-acetylphenylalanine |
| **Amino Acid** | Phenylalanine and Tyrosine Metabolism | N-acetyltyrosine |
| **Amino Acid** | Phenylalanine and Tyrosine Metabolism | N-formylphenylalanine |
| **Amino Acid** | Phenylalanine and Tyrosine Metabolism | p-cresol |
| **Amino Acid** | Phenylalanine and Tyrosine Metabolism | p-cresol sulfate |
| **Amino Acid** | Phenylalanine and Tyrosine Metabolism | phenethylamine |
| **Amino Acid** | Phenylalanine and Tyrosine Metabolism | phenylacetate |
| **Amino Acid** | Phenylalanine and Tyrosine Metabolism | phenylalanine |
| **Amino Acid** | Phenylalanine and Tyrosine Metabolism | phenyllactate (PLA) |
| **Amino Acid** | Phenylalanine and Tyrosine Metabolism | phenylpyruvate |
| **Amino Acid** | Phenylalanine and Tyrosine Metabolism | tyramine |
| **Amino Acid** | Phenylalanine and Tyrosine Metabolism | tyrosine |
| **Amino Acid** | Phenylalanine and Tyrosine Metabolism | valerylphenylalanine |
| **Amino Acid** | Polyamine Metabolism | 4-acetamidobutanoate |
| **Amino Acid** | Polyamine Metabolism | acisoga |
| **Amino Acid** | Polyamine Metabolism | N-acetylputrescine |
| **Amino Acid** | Polyamine Metabolism | spermidine |
| **Amino Acid** | Tryptophan Metabolism | 2-aminophenol |
| **Amino Acid** | Tryptophan Metabolism | indole-3-carboxylic acid |
| **Amino Acid** | Tryptophan Metabolism | indoleacetate |
| **Amino Acid** | Tryptophan Metabolism | indolelactate |
| **Amino Acid** | Tryptophan Metabolism | indolepropionate |
| **Amino Acid** | Tryptophan Metabolism | kynurenate |
| **Amino Acid** | Tryptophan Metabolism | N-acetyltryptophan |
| **Amino Acid** | Tryptophan Metabolism | picolinate |
| **Amino Acid** | Tryptophan Metabolism | serotonin |

| | | |
|---|---|---|
| **Amino Acid** | Tryptophan Metabolism | thioproline |
| **Amino Acid** | Tryptophan Metabolism | tryptamine |
| **Amino Acid** | Tryptophan Metabolism | tryptophan |
| **Amino Acid** | Urea cycle; Arginine and Proline Metabolism | arginine |
| **Amino Acid** | Urea cycle; Arginine and Proline Metabolism | citrulline |
| **Amino Acid** | Urea cycle; Arginine and Proline Metabolism | dimethylarginine (ADMA + SDMA) |
| **Amino Acid** | Urea cycle; Arginine and Proline Metabolism | homocitrulline |
| **Amino Acid** | Urea cycle; Arginine and Proline Metabolism | hydroxyproline |
| **Amino Acid** | Urea cycle; Arginine and Proline Metabolism | N-acetylarginine |
| **Amino Acid** | Urea cycle; Arginine and Proline Metabolism | N-acetylcitrulline |
| **Amino Acid** | Urea cycle; Arginine and Proline Metabolism | N-acetylproline |
| **Amino Acid** | Urea cycle; Arginine and Proline Metabolism | N-alpha-acetylornithine |
| **Amino Acid** | Urea cycle; Arginine and Proline Metabolism | N-delta-acetylornithine |
| **Amino Acid** | Urea cycle; Arginine and Proline Metabolism | N-methylproline |
| **Amino Acid** | Urea cycle; Arginine and Proline Metabolism | ornithine |
| **Amino Acid** | Urea cycle; Arginine and Proline Metabolism | proline |
| **Carbohydrate** | Advanced Glycation End-product | N6-carboxymethyllysine |
| **Carbohydrate** | Aminosugar Metabolism | erythronate* |
| **Carbohydrate** | Aminosugar Metabolism | glucuronate |
| **Carbohydrate** | Aminosugar Metabolism | N-acetyl-beta-glucosaminylamine |
| **Carbohydrate** | Aminosugar Metabolism | N-acetylglucosamine/N-acetylgalactosamine |
| **Carbohydrate** | Aminosugar Metabolism | N-acetylglucosaminylasparagine |
| **Carbohydrate** | Aminosugar Metabolism | N-acetylmuramate |
| **Carbohydrate** | Aminosugar Metabolism | N-acetylneuraminate |
| **Carbohydrate** | Fructose, Mannose and Galactose Metabolism | fructose |
| **Carbohydrate** | Fructose, Mannose and Galactose Metabolism | galactonate |
| **Carbohydrate** | Fructose, Mannose and Galactose Metabolism | mannitol/sorbitol |
| **Carbohydrate** | Fructose, Mannose and Galactose Metabolism | mannose |
| **Carbohydrate** | Glycogen Metabolism | maltose |
| **Carbohydrate** | Glycolysis, Gluconeogenesis, and Pyruvate Metabolism | glucose |
| **Carbohydrate** | Glycolysis, Gluconeogenesis, and Pyruvate Metabolism | glycerate |
| **Carbohydrate** | Glycolysis, Gluconeogenesis, and Pyruvate Metabolism | lactate |
| **Carbohydrate** | Pentose Metabolism | 2-deoxyribose |
| **Carbohydrate** | Pentose Metabolism | arabinose |
| **Carbohydrate** | Pentose Metabolism | arabitol/xylitol |
| **Carbohydrate** | Pentose Metabolism | arabonate/xylonate |
| **Carbohydrate** | Pentose Metabolism | fucose |
| **Carbohydrate** | Pentose Metabolism | ribitol |
| **Carbohydrate** | Pentose Metabolism | ribonate (ribonolactone) |
| **Carbohydrate** | Pentose Metabolism | ribulose/xylulose |
| **Carbohydrate** | Pentose Metabolism | sedoheptulose |
| **Carbohydrate** | Pentose Metabolism | xylose |
| **Cofactors and Vitamins** | Ascorbate and Aldarate Metabolism | threonate |

| | | |
|---|---|---|
| **Cofactors and Vitamins** | Biotin Metabolism | biocytin |
| **Cofactors and Vitamins** | Biotin Metabolism | biotin |
| **Cofactors and Vitamins** | Hemoglobin and Porphyrin Metabolism | bilirubin |
| **Cofactors and Vitamins** | Hemoglobin and Porphyrin Metabolism | biliverdin |
| **Cofactors and Vitamins** | Hemoglobin and Porphyrin Metabolism | D-urobilin |
| **Cofactors and Vitamins** | Hemoglobin and Porphyrin Metabolism | L-urobilin |
| **Cofactors and Vitamins** | Nicotinate and Nicotinamide Metabolism | 6-hydroxynicotinate |
| **Cofactors and Vitamins** | Nicotinate and Nicotinamide Metabolism | nicotinamide |
| **Cofactors and Vitamins** | Nicotinate and Nicotinamide Metabolism | nicotinamide riboside |
| **Cofactors and Vitamins** | Nicotinate and Nicotinamide Metabolism | nicotinate |
| **Cofactors and Vitamins** | Nicotinate and Nicotinamide Metabolism | nicotinate ribonucleoside |
| **Cofactors and Vitamins** | Nicotinate and Nicotinamide Metabolism | trigonelline (N'-methylnicotinate) |
| **Cofactors and Vitamins** | Pantothenate and CoA Metabolism | pantothenate (Vitamin B5) |
| **Cofactors and Vitamins** | Pterin Metabolism | pterin |
| **Cofactors and Vitamins** | Riboflavin Metabolism | riboflavin (Vitamin B2) |
| **Cofactors and Vitamins** | Thiamine Metabolism | 5-(2-Hydroxyethyl)-4-methylthiazole |
| **Cofactors and Vitamins** | Thiamine Metabolism | thiamin (Vitamin B1) |
| **Cofactors and Vitamins** | Tocopherol Metabolism | alpha-tocopherol |
| **Cofactors and Vitamins** | Tocopherol Metabolism | alpha-tocotrienol |
| **Cofactors and Vitamins** | Tocopherol Metabolism | gamma-tocopherol/beta-tocopherol |
| **Cofactors and Vitamins** | Tocopherol Metabolism | gamma-tocotrienol |
| **Cofactors and Vitamins** | Vitamin B6 Metabolism | pyridoxal |
| **Cofactors and Vitamins** | Vitamin B6 Metabolism | pyridoxamine |
| **Cofactors and Vitamins** | Vitamin B6 Metabolism | pyridoxate |
| **Cofactors and Vitamins** | Vitamin B6 Metabolism | pyridoxine (Vitamin B6) |
| **Energy** | Oxidative Phosphorylation | phosphate |
| **Energy** | TCA Cycle | 2-methylcitrate/homocitrate |
| **Energy** | TCA Cycle | alpha-ketoglutarate |
| **Energy** | TCA Cycle | citrate |
| **Energy** | TCA Cycle | fumarate |

| | | |
|---|---|---|
| **Energy** | TCA Cycle | malate |
| **Energy** | TCA Cycle | succinate |
| **Energy** | TCA Cycle | tricarballylate |
| **Lipid** | Carnitine Metabolism | carnitine |
| **Lipid** | Carnitine Metabolism | deoxycarnitine |
| **Lipid** | Diacylglycerol | 1-oleoyl-3-linoleoyl-glycerol (18:1/18:2) |
| **Lipid** | Diacylglycerol | 1-palmitoyl-3-linoleoyl-glycerol (16:0/18:2)* |
| **Lipid** | Endocannabinoid | linoleoyl ethanolamide |
| **Lipid** | Endocannabinoid | oleoyl ethanolamide |
| **Lipid** | Endocannabinoid | palmitoyl ethanolamide |
| **Lipid** | Endocannabinoid | stearoyl ethanolamide |
| **Lipid** | Fatty Acid Metabolism (also BCAA Metabolism) | propionylglycine (C3) |
| **Lipid** | Fatty Acid Synthesis | malonate |
| **Lipid** | Fatty Acid, Amide | linoleamide (18:2n6) |
| **Lipid** | Fatty Acid, Branched | 13-methylmyristate |
| **Lipid** | Fatty Acid, Branched | 17-methylstearate |
| **Lipid** | Fatty Acid, Branched | methylpalmitate (15 or 2) |
| **Lipid** | Fatty Acid, Branched | pristanate |
| **Lipid** | Fatty Acid, Dicarboxylate | 1,11-undecanedicarboxylate |
| **Lipid** | Fatty Acid, Dicarboxylate | 2-hydroxyglutarate |
| **Lipid** | Fatty Acid, Dicarboxylate | 3-carboxyadipate |
| **Lipid** | Fatty Acid, Dicarboxylate | 3-methyladipate |
| **Lipid** | Fatty Acid, Dicarboxylate | 3-methylglutarate/2-methylglutarate |
| **Lipid** | Fatty Acid, Dicarboxylate | adipate |
| **Lipid** | Fatty Acid, Dicarboxylate | azelate (nonanedioate; C9) |
| **Lipid** | Fatty Acid, Dicarboxylate | dimethylmalonic acid |
| **Lipid** | Fatty Acid, Dicarboxylate | dodecanedioate (C12) |
| **Lipid** | Fatty Acid, Dicarboxylate | hexadecanedioate (C16) |
| **Lipid** | Fatty Acid, Dicarboxylate | maleate |
| **Lipid** | Fatty Acid, Dicarboxylate | octadecanedioate (C18) |
| **Lipid** | Fatty Acid, Dicarboxylate | sebacate (decanedioate) |
| **Lipid** | Fatty Acid, Dicarboxylate | suberate (octanedioate) |
| **Lipid** | Fatty Acid, Dicarboxylate | undecanedioate |
| **Lipid** | Fatty Acid, Dihydroxy | 12,13-DiHOME |
| **Lipid** | Fatty Acid, Dihydroxy | 9,10-DiHOME |
| **Lipid** | Fatty Acid, Monohydroxy | 10-hydroxystearate |
| **Lipid** | Fatty Acid, Monohydroxy | 13-HODE + 9-HODE |
| **Lipid** | Fatty Acid, Monohydroxy | 2-hydroxydecanoate |
| **Lipid** | Fatty Acid, Monohydroxy | 2-hydroxypalmitate |
| **Lipid** | Fatty Acid, Monohydroxy | 3-hydroxymyristate |
| **Lipid** | Fatty Acid, Monohydroxy | 3-hydroxystearate |
| **Lipid** | Fatty Acid, Monohydroxy | 5-hydroxyhexanoate |
| **Lipid** | Glycerolipid Metabolism | glycerol |
| **Lipid** | Glycerolipid Metabolism | glycerol 3-phosphate |
| **Lipid** | Glycerolipid Metabolism | glycerophosphoglycerol |

| | | |
|---|---|---|
| **Lipid** | Inositol Metabolism | myo-inositol |
| **Lipid** | Ketone Bodies | 3-hydroxybutyrate (BHBA) |
| **Lipid** | Long Chain Fatty Acid | 10-heptadecenoate (17:1n7) |
| **Lipid** | Long Chain Fatty Acid | arachidate (20:0) |
| **Lipid** | Long Chain Fatty Acid | eicosenoate (20:1n9 or 1n11) |
| **Lipid** | Long Chain Fatty Acid | erucate (22:1n9) |
| **Lipid** | Long Chain Fatty Acid | margarate (17:0) |
| **Lipid** | Long Chain Fatty Acid | myristate (14:0) |
| **Lipid** | Long Chain Fatty Acid | myristoleate (14:1n5) |
| **Lipid** | Long Chain Fatty Acid | nonadecanoate (19:0) |
| **Lipid** | Long Chain Fatty Acid | oleate/vaccenate (18:1) |
| **Lipid** | Long Chain Fatty Acid | palmitate (16:0) |
| **Lipid** | Long Chain Fatty Acid | palmitoleate (16:1n7) |
| **Lipid** | Long Chain Fatty Acid | stearate (18:0) |
| **Lipid** | Lysolipid | 1-palmitoyl-GPC (16:0) |
| **Lipid** | Lysolipid | 1-palmitoyl-GPE (16:0) |
| **Lipid** | Lysolipid | 1-palmitoyl-GPG (16:0)* |
| **Lipid** | Lysolipid | 1-stearoyl-GPC (18:0) |
| **Lipid** | Lysolipid | 1-stearoyl-GPE (18:0) |
| **Lipid** | Lysoplasmalogen | 1-(1-enyl-stearoyl)-GPE (P-18:0)* |
| **Lipid** | Medium Chain Fatty Acid | 5-dodecenoate (12:1n7) |
| **Lipid** | Medium Chain Fatty Acid | caprate (10:0) |
| **Lipid** | Medium Chain Fatty Acid | caproate (6:0) |
| **Lipid** | Medium Chain Fatty Acid | caprylate (8:0) |
| **Lipid** | Medium Chain Fatty Acid | heptanoate (7:0) |
| **Lipid** | Medium Chain Fatty Acid | laurate (12:0) |
| **Lipid** | Mevalonate Metabolism | 3-hydroxy-3-methylglutarate |
| **Lipid** | Mevalonate Metabolism | mevalonate |
| **Lipid** | Monoacylglycerol | 1-linoleoylglycerol (18:2) |
| **Lipid** | Monoacylglycerol | 1-palmitoylglycerol (16:0) |
| **Lipid** | Phospholipid Metabolism | 1-oleoyl-2-linoleoyl-GPC (18:1/18:2)* |
| **Lipid** | Phospholipid Metabolism | 1-palmitoyl-2-linoleoyl-GPC (16:0/18:2) |
| **Lipid** | Phospholipid Metabolism | 1-palmitoyl-2-oleoyl-GPC (16:0/18:1) |
| **Lipid** | Phospholipid Metabolism | choline |
| **Lipid** | Phospholipid Metabolism | glycerophosphoethanolamine |
| **Lipid** | Phospholipid Metabolism | glycerophosphoinositol* |
| **Lipid** | Phospholipid Metabolism | phosphocholine |
| **Lipid** | Phospholipid Metabolism | trimethylamine N-oxide |
| **Lipid** | Polyunsaturated Fatty Acid (n3 and n6) | arachidonate (20:4n6) |
| **Lipid** | Polyunsaturated Fatty Acid (n3 and n6) | dihomolinoleate (20:2n6) |
| **Lipid** | Polyunsaturated Fatty Acid (n3 and n6) | dihomolinolenate (20:3n3 or 3n6) |
| **Lipid** | Polyunsaturated Fatty Acid (n3 and n6) | docosadienoate (22:2n6) |
| **Lipid** | Polyunsaturated Fatty Acid (n3 and n6) | docosahexaenoate (DHA; 22:6n3) |
| **Lipid** | Polyunsaturated Fatty Acid (n3 and n6) | docosapentaenoate (DPA; 22:5n3) |
| **Lipid** | Polyunsaturated Fatty Acid (n3 and n6) | eicosapentaenoate (EPA; 20:5n3) |

| | | |
|---|---|---|
| **Lipid** | Polyunsaturated Fatty Acid (n3 and n6) | linoleate (18:2n6) |
| **Lipid** | Polyunsaturated Fatty Acid (n3 and n6) | linolenate (18:3n3 or 3n6) |
| **Lipid** | Primary Bile Acid Metabolism | cholate |
| **Lipid** | Primary Bile Acid Metabolism | glycochenodeoxycholate |
| **Lipid** | Primary Bile Acid Metabolism | glycocholate |
| **Lipid** | Secondary Bile Acid Metabolism | 3b-hydroxy-5-cholenoic acid |
| **Lipid** | Secondary Bile Acid Metabolism | 6-oxolithocholate |
| **Lipid** | Secondary Bile Acid Metabolism | dehydrolithocholate |
| **Lipid** | Secondary Bile Acid Metabolism | deoxycholate |
| **Lipid** | Secondary Bile Acid Metabolism | glycodeoxycholate |
| **Lipid** | Secondary Bile Acid Metabolism | glycolithocholate sulfate* |
| **Lipid** | Secondary Bile Acid Metabolism | hyocholate |
| **Lipid** | Secondary Bile Acid Metabolism | lithocholate |
| **Lipid** | Short Chain Fatty Acid | valerate (5:0) |
| **Lipid** | Sphingolipid Metabolism | 3-ketosphinganine |
| **Lipid** | Sphingolipid Metabolism | N-palmitoyl-sphinganine (d18:0/16:0) |
| **Lipid** | Sphingolipid Metabolism | N-palmitoyl-sphingosine (d18:1/16:0) |
| **Lipid** | Sphingolipid Metabolism | palmitoyl sphingomyelin (d18:1/16:0) |
| **Lipid** | Sphingolipid Metabolism | phytosphingosine |
| **Lipid** | Sphingolipid Metabolism | sphinganine |
| **Lipid** | Sphingolipid Metabolism | sphingosine |
| **Lipid** | Sterol | 4-cholesten-3-one |
| **Lipid** | Sterol | beta-sitosterol |
| **Lipid** | Sterol | campesterol |
| **Lipid** | Sterol | cholesterol |
| **Lipid** | Sterol | coprostanol |
| **Lipid** | Sterol | lanosterol |
| **Nucleotide** | Purine and Pyrimidine Metabolism | methylphosphate |
| **Nucleotide** | Purine Metabolism, (Hypo)Xanthine/Inosine containing | 2'-deoxyinosine |
| **Nucleotide** | Purine Metabolism, (Hypo)Xanthine/Inosine containing | hypoxanthine |
| **Nucleotide** | Purine Metabolism, (Hypo)Xanthine/Inosine containing | inosine |
| **Nucleotide** | Purine Metabolism, (Hypo)Xanthine/Inosine containing | urate |
| **Nucleotide** | Purine Metabolism, (Hypo)Xanthine/Inosine containing | xanthine |
| **Nucleotide** | Purine Metabolism, Adenine containing | 1-methyladenine |
| **Nucleotide** | Purine Metabolism, Adenine containing | 2'-deoxyadenosine |
| **Nucleotide** | Purine Metabolism, Adenine containing | adenine |
| **Nucleotide** | Purine Metabolism, Adenine containing | adenosine |
| **Nucleotide** | Purine Metabolism, Adenine containing | N6-dimethylallyladenine |
| **Nucleotide** | Purine Metabolism, Guanine containing | 2'-deoxyguanosine |
| **Nucleotide** | Purine Metabolism, Guanine containing | 7-methylguanine |
| **Nucleotide** | Purine Metabolism, Guanine containing | 8-hydroxyguanine |
| **Nucleotide** | Purine Metabolism, Guanine containing | guanine |
| **Nucleotide** | Purine Metabolism, Guanine containing | guanosine |

| | | |
|---|---|---|
| **Nucleotide** | Pyrimidine Metabolism, Cytidine containing | 2'-deoxycytidine |
| **Nucleotide** | Pyrimidine Metabolism, Cytidine containing | cytidine |
| **Nucleotide** | Pyrimidine Metabolism, Cytidine containing | cytosine |
| **Nucleotide** | Pyrimidine Metabolism, Orotate containing | dihydroorotate |
| **Nucleotide** | Pyrimidine Metabolism, Orotate containing | N-carbamoylaspartate |
| **Nucleotide** | Pyrimidine Metabolism, Orotate containing | orotate |
| **Nucleotide** | Pyrimidine Metabolism, Thymine containing | thymidine |
| **Nucleotide** | Pyrimidine Metabolism, Thymine containing | thymine |
| **Nucleotide** | Pyrimidine Metabolism, Uracil containing | 2'-deoxyuridine |
| **Nucleotide** | Pyrimidine Metabolism, Uracil containing | 3-ureidopropionate |
| **Nucleotide** | Pyrimidine Metabolism, Uracil containing | 4-ureidobutyrate |
| **Nucleotide** | Pyrimidine Metabolism, Uracil containing | 5-methyluridine (ribothymidine) |
| **Nucleotide** | Pyrimidine Metabolism, Uracil containing | beta-alanine |
| **Nucleotide** | Pyrimidine Metabolism, Uracil containing | pseudouridine |
| **Nucleotide** | Pyrimidine Metabolism, Uracil containing | uracil |
| **Nucleotide** | Pyrimidine Metabolism, Uracil containing | uridine |
| **Peptide** | Dipeptide | alanylleucine |
| **Peptide** | Dipeptide | glycylisoleucine |
| **Peptide** | Dipeptide | glycylleucine |
| **Peptide** | Dipeptide | glycylvaline |
| **Peptide** | Dipeptide | isoleucylglycine |
| **Peptide** | Dipeptide | leucylalanine |
| **Peptide** | Dipeptide | leucylglutamine* |
| **Peptide** | Dipeptide | leucylglycine |
| **Peptide** | Dipeptide | phenylalanylalanine |
| **Peptide** | Dipeptide | phenylalanylglycine |
| **Peptide** | Dipeptide | threonylphenylalanine |
| **Peptide** | Dipeptide | tryptophylglycine |
| **Peptide** | Dipeptide | valylglutamine |
| **Peptide** | Dipeptide | valylglycine |
| **Peptide** | Dipeptide | valylleucine |
| **Peptide** | Dipeptide Derivative | anserine |
| **Peptide** | Gamma-glutamyl Amino Acid | gamma-glutamylalanine |
| **Peptide** | Gamma-glutamyl Amino Acid | gamma-glutamyl-epsilon-lysine |
| **Peptide** | Gamma-glutamyl Amino Acid | gamma-glutamylglutamate |
| **Peptide** | Gamma-glutamyl Amino Acid | gamma-glutamylglycine |
| **Peptide** | Gamma-glutamyl Amino Acid | gamma-glutamylisoleucine* |
| **Peptide** | Gamma-glutamyl Amino Acid | gamma-glutamylleucine |
| **Peptide** | Gamma-glutamyl Amino Acid | gamma-glutamylmethionine |
| **Peptide** | Gamma-glutamyl Amino Acid | gamma-glutamylphenylalanine |
| **Peptide** | Gamma-glutamyl Amino Acid | gamma-glutamyltyrosine |
| **Xenobiotics** | Benzoate Metabolism | 2,4,6-trihydroxybenzoate |
| **Xenobiotics** | Benzoate Metabolism | 3,4-dihydroxybenzoate |
| **Xenobiotics** | Benzoate Metabolism | benzoate |
| **Xenobiotics** | Chemical | 1,3-propanediol |
| **Xenobiotics** | Chemical | 2-oxo-1-pyrrolidinepropionate |

| | | |
|---|---|---|
| **Xenobiotics** | Chemical | 3-hydroxypyridine |
| **Xenobiotics** | Chemical | diethanolamine |
| **Xenobiotics** | Chemical | diglycerol |
| **Xenobiotics** | Chemical | ectoine |
| **Xenobiotics** | Chemical | N-methylpipecolate |
| **Xenobiotics** | Chemical | N-propionylmethionine |
| **Xenobiotics** | Chemical | succinimide |
| **Xenobiotics** | Chemical | sulfate* |
| **Xenobiotics** | Drug | N-carbamylglutamate |
| **Xenobiotics** | Food Component/Plant | 1-methyl-beta-carboline-3-carboxylic acid |
| **Xenobiotics** | Food Component/Plant | 2,3-dihydroxyisovalerate |
| **Xenobiotics** | Food Component/Plant | 2-isopropylmalate |
| **Xenobiotics** | Food Component/Plant | 2-keto-3-deoxy-gluconate |
| **Xenobiotics** | Food Component/Plant | 2-oxindole-3-acetate |
| **Xenobiotics** | Food Component/Plant | 2-piperidinone |
| **Xenobiotics** | Food Component/Plant | beta-guanidinopropanoate |
| **Xenobiotics** | Food Component/Plant | caffeate |
| **Xenobiotics** | Food Component/Plant | diaminopimelate |
| **Xenobiotics** | Food Component/Plant | dihydroferulic acid |
| **Xenobiotics** | Food Component/Plant | dipicolinate |
| **Xenobiotics** | Food Component/Plant | enterolactone |
| **Xenobiotics** | Food Component/Plant | erythrose |
| **Xenobiotics** | Food Component/Plant | ferulate |
| **Xenobiotics** | Food Component/Plant | galacturonate |
| **Xenobiotics** | Food Component/Plant | gluconate |
| **Xenobiotics** | Food Component/Plant | indolin-2-one |
| **Xenobiotics** | Food Component/Plant | levulinate (4-oxovalerate) |
| **Xenobiotics** | Food Component/Plant | nicotianamine |
| **Xenobiotics** | Food Component/Plant | pheophorbide A |
| **Xenobiotics** | Food Component/Plant | phytanate |
| **Xenobiotics** | Food Component/Plant | piperidine |
| **Xenobiotics** | Food Component/Plant | piperine |
| **Xenobiotics** | Food Component/Plant | pyrraline |
| **Xenobiotics** | Food Component/Plant | quinate |
| **Xenobiotics** | Food Component/Plant | sitostanol |
| **Xenobiotics** | Food Component/Plant | solanidine |
| **Xenobiotics** | Food Component/Plant | stachydrine |
| **Xenobiotics** | Food Component/Plant | tyrosol |
| **Xenobiotics** | Xanthine Metabolism | 1,3,7-trimethylurate |
| **Xenobiotics** | Xanthine Metabolism | 1,3-dimethylurate |
| **Xenobiotics** | Xanthine Metabolism | 1,7-dimethylurate |
| **Xenobiotics** | Xanthine Metabolism | 3,7-dimethylurate |
| **Xenobiotics** | Xanthine Metabolism | 5-acetylamino-6-amino-3-methyluracil |
| **Xenobiotics** | Xanthine Metabolism | caffeine |
| **Xenobiotics** | Xanthine Metabolism | paraxanthine |

| | | |
|---|---|---|
| **Xenobiotics** | Xanthine Metabolism | theophylline |
